# Supplementary material for: Predicting the Prognostic Value of POLI Expression in Different Cancers via a Machine Learning Approach
Source: Int J Mol Sci. 2022 Aug 2;23(15):8571. doi: 10.3390/ijms23158571 (PMC9369001; doi:10.3390/ijms23158571)
Supplement: Supplementary file 1 [file ijms-23-08571-s001.zip › ijms-1804944-supplementary.pdf]

Supplementary Material

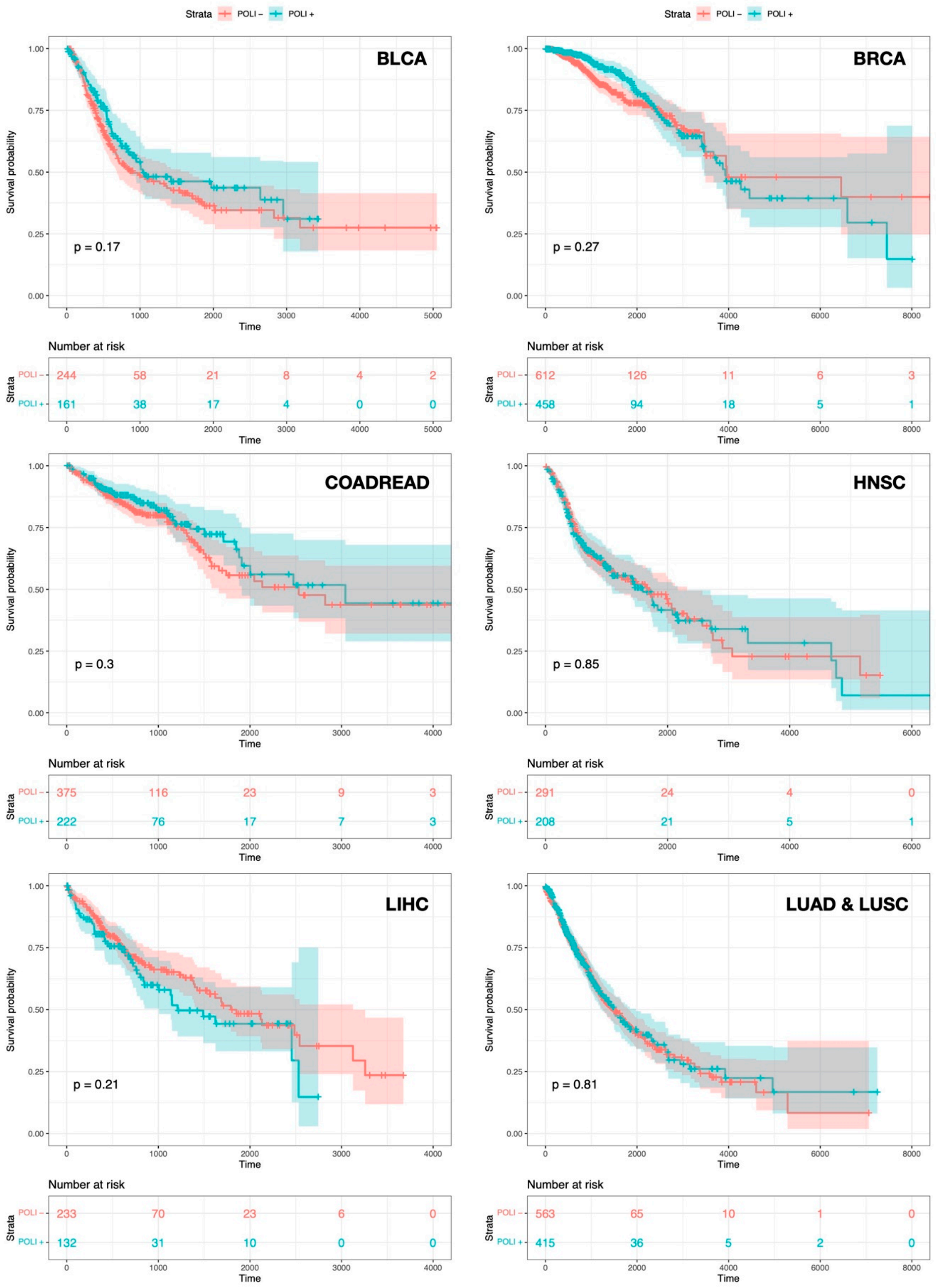

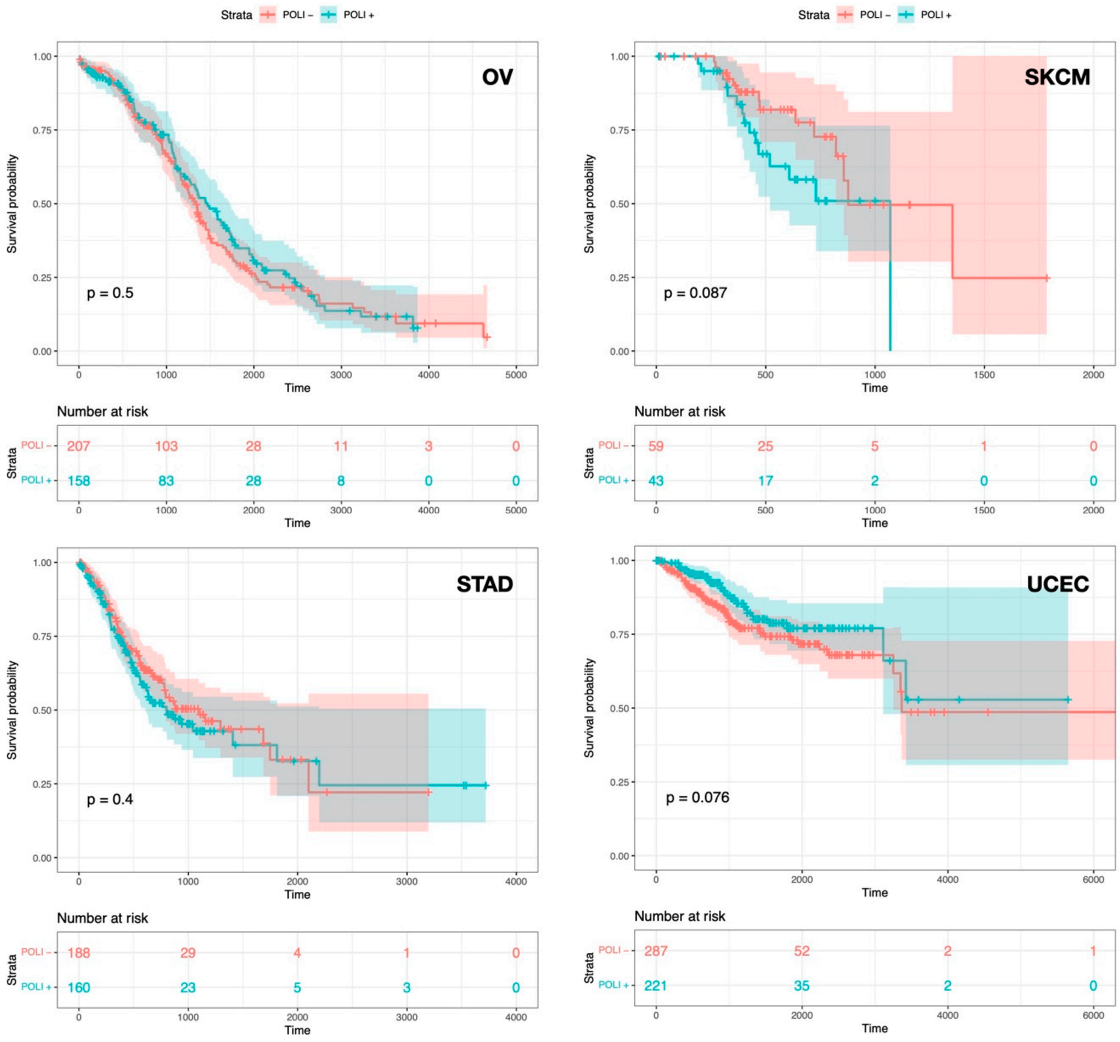

Figure S1. Prognostic value of POLI in BLCA, BRAC, COADREAD, HNSC, LIHC, LUAD & LUSC, OV, SKCM, STAD, and UCEC.

The correlation between genes and POLI were calculated again using Spearman's rank correlation. In Table S2, the ranges of Spearman correlation coefficient were slightly different from values in Table 1 via Pearson correlation. The exact analytical procedure was executed using data based on Spearman correlation. Results from Pearson correlation yielded smaller *p*-values when selected same subtypes in Group 1 and Group 2 compared with Spearman correlation.

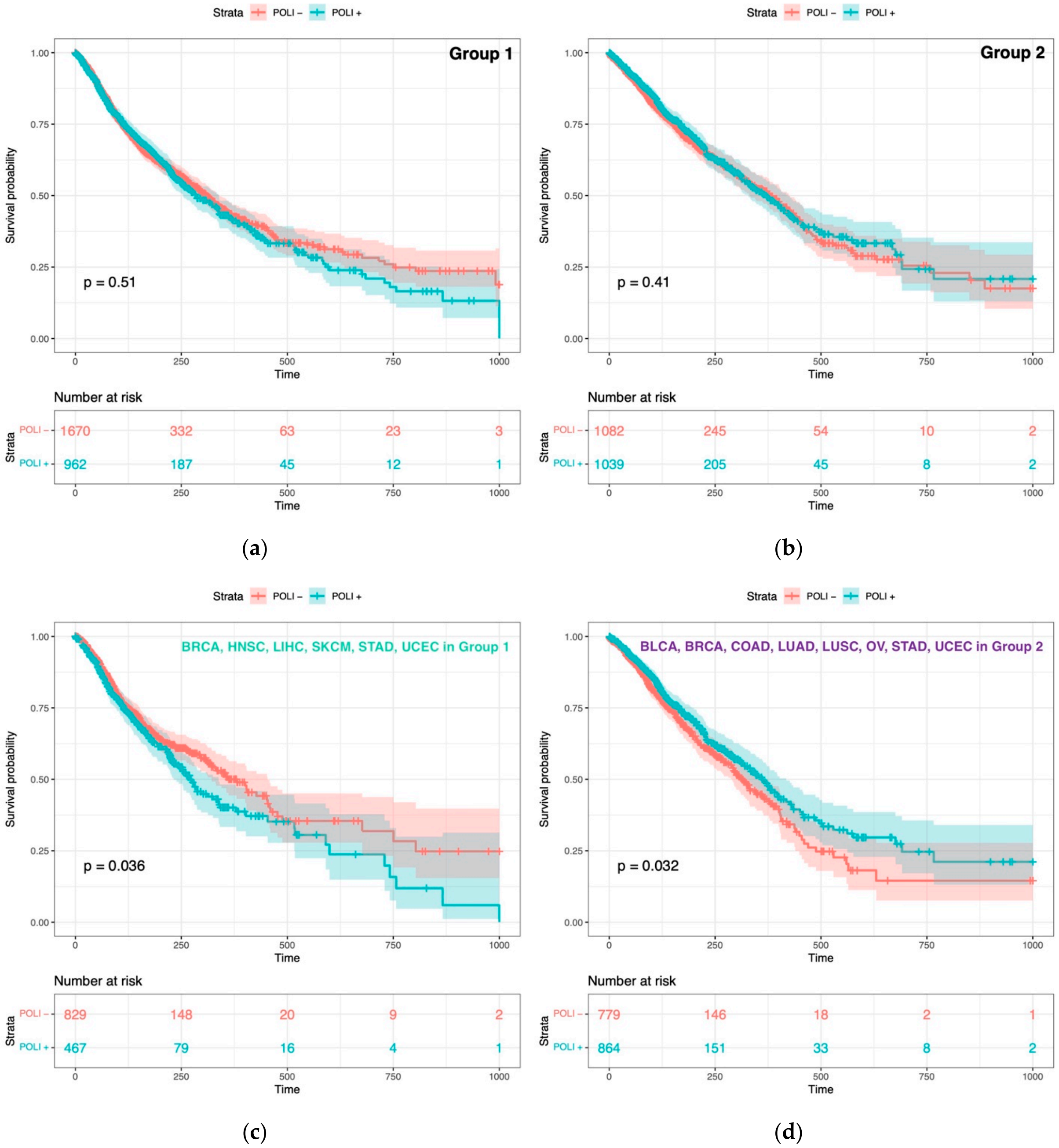

Figure S2. Reoccurrence of the prognostic value of POLI expression via Spearman correlation: (**a**) K-M plot of POLI expression for patients in Group 1; (**b**) K-M plot of POLI expression for patients in Group 2; (**c**) K-M plot of POLI expression for patients in BRAC, HNSC, LIHC, SKCM, STAD, and UCEC from Group 1; (**d**) K-M plot of POLI expression for patients in BLCA, BRAC, COADREAD, LUAD, LUSC, OV, STAD, and UCEC from Group 2.

Gradient Boosting (GB) and Neural networks (NN) were applied to data via Pearson correlation using the identical setting from RFC in this study (Figures S4 and S5). Either method yielded larger p-value compared to the results using Random Forest Classifier in Figure 2. NN model was implemented in TensorFlow with four layers and half data drop out in each layer. The accuracy of predicting CESC and PAAD in testing dataset was 98%. Gradient boosting classifier was implemented in *scikit-learn* module with 100 estimators and 0.5 learning rate. The accuracy of predicting CESC and PAAD in testing dataset was 100%. Similarly, but less successfully, two other classifiers were roughly clustered patients with POLI as a negative prognostic factor in Group 1 or a positive prognostic factor in Group 2.

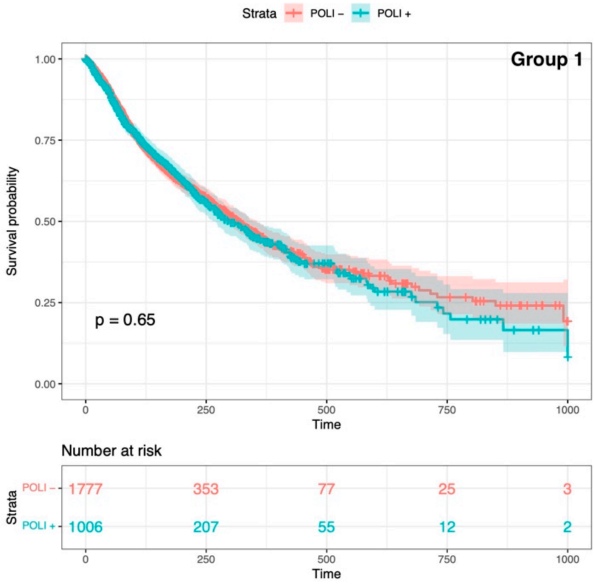

(**a**)

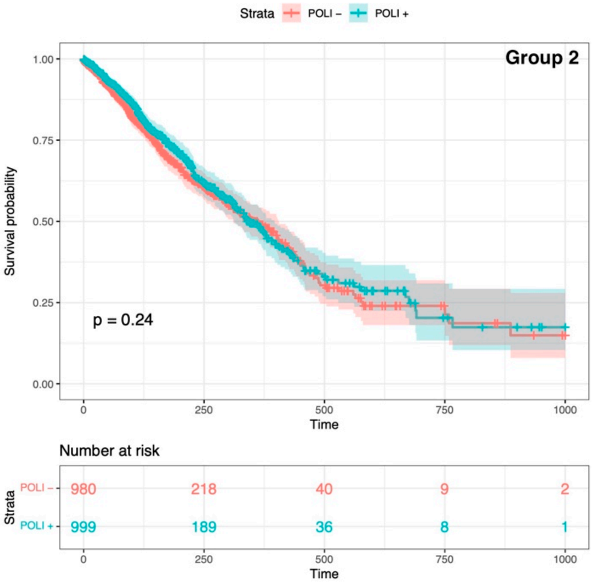

(**b**)

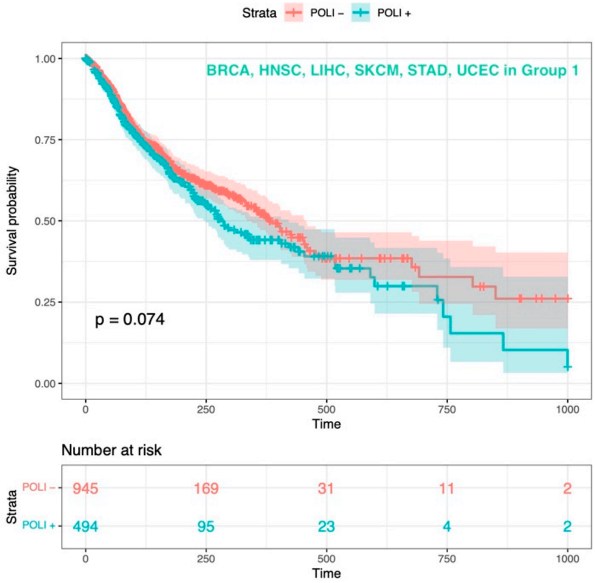

(**c**)

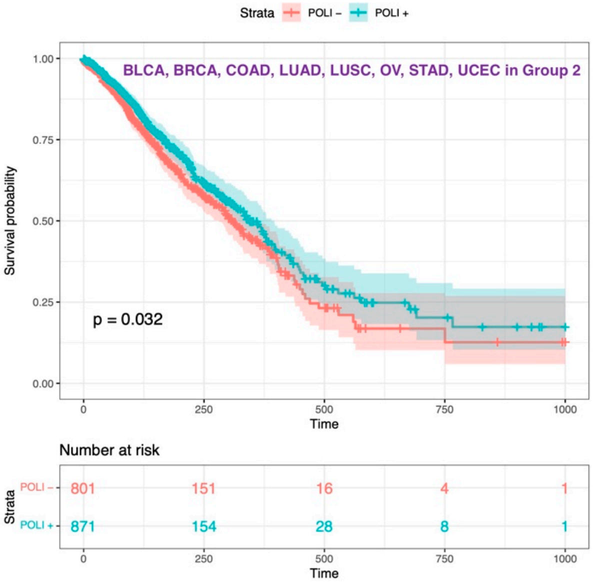

(**d**)

Figure S3. The prognostic value of POLI expression in Group 1 and Group 2 via gradient boosting classifier: (**a**) K-M plot of POLI expression for patients in Group 1; (**b**) K-M plot of POLI expression for patients in Group 2; (**c**) K-M plot of POLI expression for patients in BRAC, HNSC, LIHC, SKCM, STAD, and UCEC from Group 1; (**d**) K-M plot of POLI expression for patients in BLCA, BRAC, COADREAD, LUAD, LUSC, OV, STAD, and UCEC from Group 2.

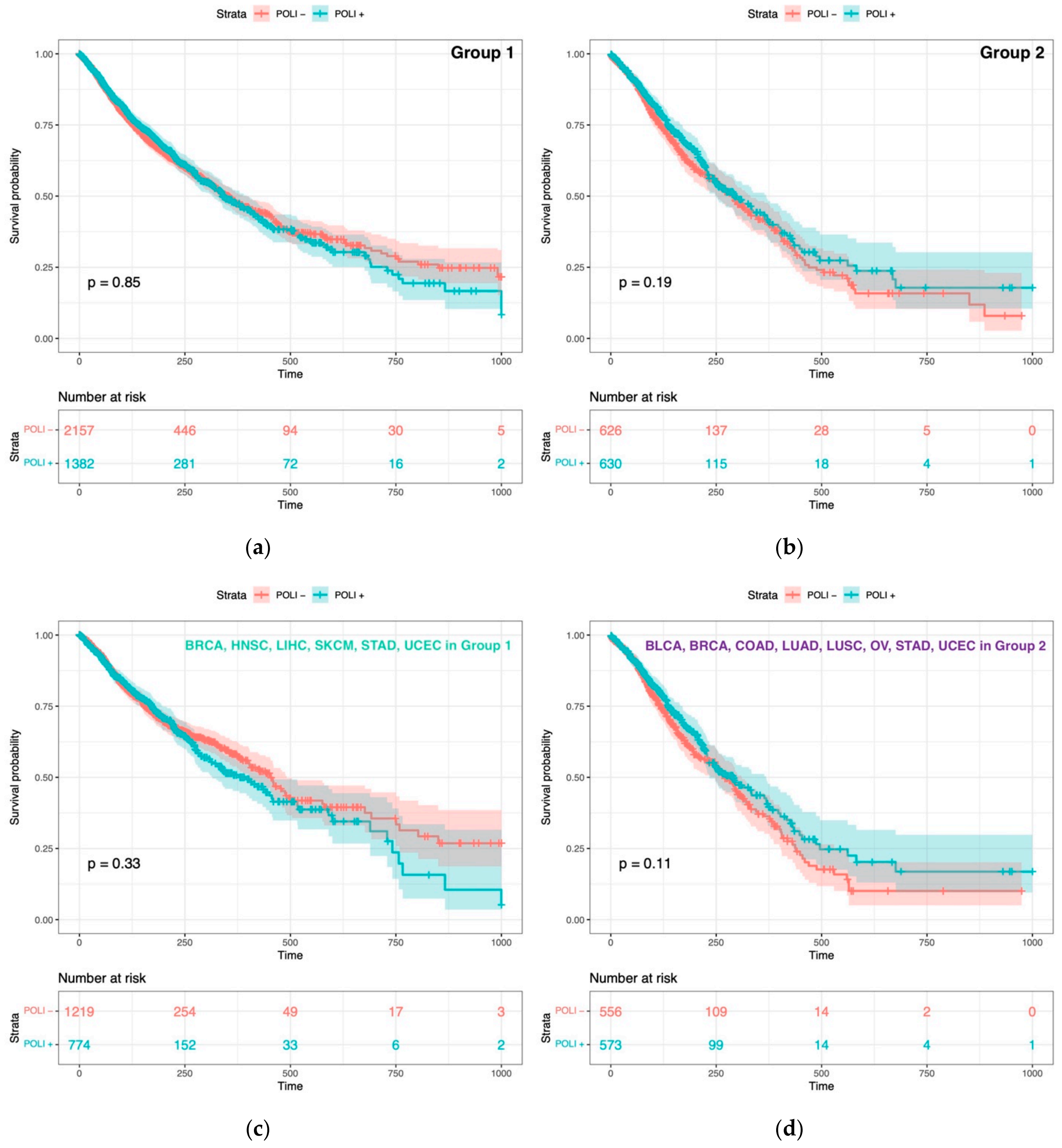

Figure S4. The prognostic value of POLI expression in Group 1 and Group 2 via NN classifier: (**a**) K-M plot of POLI expression for patients in Group 1; (**b**) K-M plot of POLI expression for patients in Group 2; (**c**) K-M plot of POLI expression for patients in BRAC, HNSC, LIHC, SKCM, STAD, and UCEC from Group 1; (**d**) K-M plot of POLI expression for patients in BLCA, BRAC, COADREAD, LUAD, LUSC, OV, STAD, and UCEC from Group 2.

The importance of gene variables was provided in RFM. Top 100 genes were re-processed via GLASSO to reduce the size to 25 highly associated genes using gene-gene correlation matrix of CESC and PAAD, respectively. Six genes (CLPP, EDF1, RPL28, RPS15, SF3B5 and SURF2) existed in both cancer types. 25 genes formed five to six clusters in CESC or PAAD that indicates gene-gene interaction conditioning on POLI expression and potential factors regulating POLI expression (Figure 3).

In CESC, AURKAIP1 was associated to 11 genes forming an isolated cluster consisting of 13 genes. Other independent clusters contained 2 to 3 highly associated genes. In PAAD, three genes including RPS15, CLPP, and NDUFB11 were all associated to five genes, however, all of them came from the same cluster with ten genes.

CLPP, EDF1, RPL28, RPS15, SF3B5, and SURF2 were existed in CESC and PAAD regarding top 25 highly associated genes.

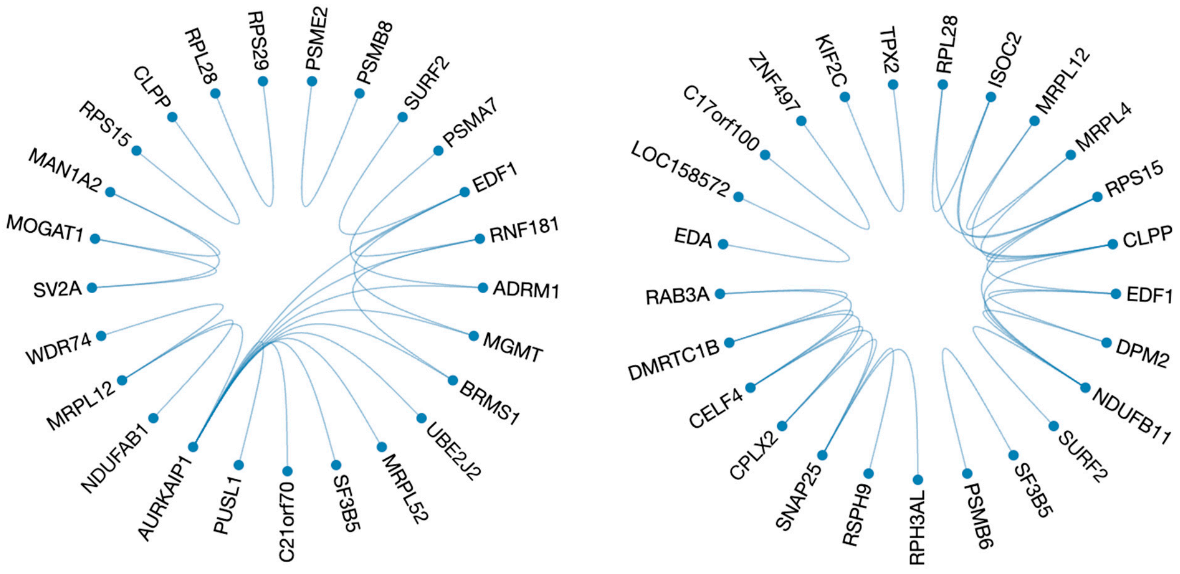

(**a**) (**b**)

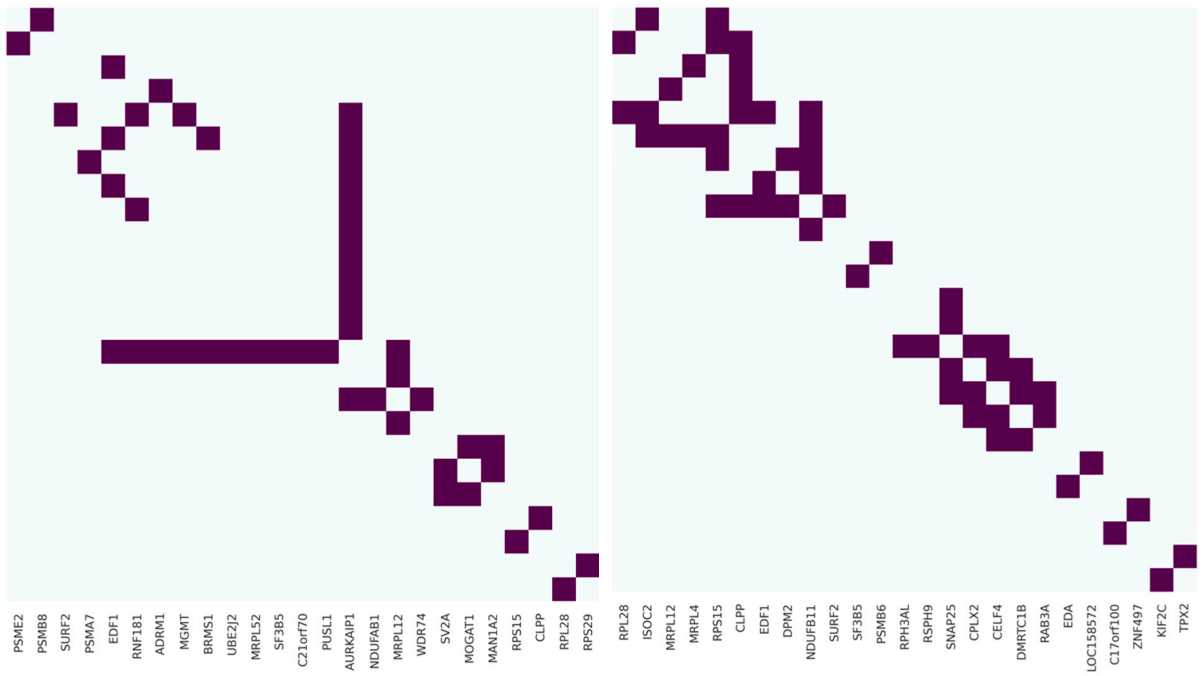

(**c**) (**d**)

Figure S5. Visualization for most correlated genes regarding POLI expression in network and heatmap layouts: (**a**) Circosplot for 25 most POLI-related genes from 100 genes returned by RFC in CESC; (**b**) Circosplot for 25 most POLI-related genes from 100 genes returned by RFC in PAAD; (**c**) heatmap layout of 25 genes in (**a**); (**d**) heatmap layout of 25 genes in (**b**).

Table S1. POLI vs other gene correlation ranges in CESC and PAAD using Spearman's rank correlation.

**Caner**	**Top 500 Positive**		**Top 500 Negative**	
	**Max**	**Min**	**Min**	**Max**
CESC	0.518 [1]	0.304	−0.409	−0.242
PAAD	0.792	0.486	−0.629	−0.402

[1] Each value indicates the Spearman's rank correlation coefficient between POLI and another gene.

Table S2. Top 100 genes assessed by RFC for the classification training task of differentiating CESC and PAAD patients.

**Gene**	**Importance**	**Gene**	**Importance**
PSMB8	0.06316943	DMRTC1B	0.0037518
PSMA7	0.05032968	ZNF497	0.00339815
UBE2S	0.04979548	NAP1L3	0.00297679
RNF181	0.04128673	CCNB2	0.002361
MRPL4	0.04051191	ECE2	0.00233863

MRPL14	0.03439445	NSUN5	0.00193941
FSIP1	0.03284802	IKZF4	0.00174226
MRPL17	0.02735727	DPM2	0.00171191
MOGAT1	0.02650861	IFI27	0.00170035
PSMB6	0.02531586	RGPD4	0.00166636
C5	0.02514696	TECTA	0.00151018
ZNF441	0.02483958	C17orf100	0.00149179
DTX3	0.02440788	RAB3A	0.00136568
RSPH9	0.02428636	MRPL37	0.00129391
WDR74	0.02252129	ZNF25	0.00128354
PCDHA2	0.0212011	TPX2	0.0012455
TMEM149	0.02046246	PCDHA3	0.00119688
AGAP4	0.02022953	LYPLA2P1	0.00117035
LOC284440	0.01957489	SALL2	0.0011319
SURF2	0.01844377	KIAA0319	0.0011106
CLPP	0.01762643	GOLGA6L5	0.00109381
RPS29	0.01727722	NDUFAB1	0.00108598
KRT8	0.01662942	CENPF	0.00105923
DNAH7	0.01639963	WDR17	0.00105264
PRELID1	0.01596565	POLD2	0.00103434
PUSL1	0.01523146	SPATA4	0.00101323
MRPL12	0.01505887	RGS4	0.00100629
CPLX2	0.01486688	MAGI2	0.00098264
AP2S1	0.01414428	PMPCA	0.00095689
LOC158572	0.01374755	MX1	0.00095484
PSME2	0.01168913	TIGD4	0.00093922
ISOC2	0.00970972	RPS15	0.00092699
GPKOW	0.00928349	KIF15	0.00091967
C21orf70	0.00873391	MGMT	0.00091269
BST2	0.00850269	KIF2C	0.00091158
KIAA1109	0.00719654	HMGA1	0.00089834
KIF20A	0.00685156	MRPL52	0.00089205
SDHAF2	0.00653688	NDUFB11	0.0008833
BRMS1	0.00630161	RPH3AL	0.00080189
LOC653501	0.00623308	ANXA2P2	0.0008009
ZNF132	0.00555305	CELF4	0.00077638
SPTB	0.00537147	EDA	0.00071937
PGAM2	0.00505307	AURKAIP1	0.00071874
EDF1	0.00483283	GJB5	0.00069368
SF3B5	0.00471939	UBE2J2	0.00069125
UQCRC1	0.0047081	SNAP25	0.00068137
ZWINT	0.00468075	TGIF2LY	0.00067734
MAN1A2	0.00411337	SV2A	0.00065265
CTXN2	0.00390633	RPL28	0.00064673
